# Quantitative molecular diagnostic assays of grain washes for *Claviceps purpurea* are correlated with visual determinations of ergot contamination

**DOI:** 10.1371/journal.pone.0173495

**Published:** 2017-03-03

**Authors:** Alexia Comte, Tom Gräfenhan, Matthew G. Links, Sean M. Hemmingsen, Tim J. Dumonceaux

**Affiliations:** 1 Agriculture and Agri-Food Canada Saskatoon Research and Development Centre, Saskatoon, Saskatchewan, Canada; 2 Grain Research Laboratory, Canadian Grain Commission, Winnipeg, Manitoba, Canada; 3 Department of Computer Science, University of Saskatchewan, Saskatoon, Saskatchewan, Canada; 4 National Research Council Canada, Saskatoon, Saskatchewan, Canada; 5 Department of Microbiology and Immunology, University of Saskatchewan, Saskatoon, Saskatchewan, Canada; 6 Department of Veterinary Microbiology, University of Saskatchewan, Saskatoon, Saskatchewan, Canada; McGill University, CANADA

## Abstract

We examined the epiphytic microbiome of cereal grain using the universal barcode chaperonin-60 (*cpn60*). Microbial community profiling of seed washes containing DNA extracts prepared from field-grown cereal grain detected sequences from a fungus identified only to Class Sordariomycetes. To identify the fungal sequence and to improve the reference database, we determined *cpn60* sequences from field-collected and reference strains of the ergot fungus, *Claviceps purpurea*. These data allowed us to identify this fungal sequence as deriving from *C*. *purpurea*, and suggested that *C*. *purpurea* DNA is readily detectable on agricultural commodities, including those for which ergot was not identified as a grading factor. To get a sense of the prevalence and level of *C*. *purpurea* DNA in cereal grains, we developed a quantitative PCR assay based on the fungal internal transcribed spacer (ITS) and applied it to 137 samples from the 2014 crop year. The amount of *Claviceps* DNA quantified correlated strongly with the proportion of ergot sclerotia identified in each grain lot, although there was evidence that non-target organisms were responsible for some false positives with the ITS-based assay. We therefore developed a *cpn60*-targeted loop-mediated isothermal amplification assay and applied it to the same grain wash samples. The time to positive displayed a significant, inverse correlation to ergot levels determined by visual ratings. These results indicate that both laboratory-based and field-adaptable molecular diagnostic assays can be used to detect and quantify pathogen load in bulk commodities using cereal grain washes.

## Introduction

*Claviceps purpurea* (Fr.) Tul. (ergot) is a fungus that infects cereal crops such as rye (*Secale cereale*), wheat (*Triticum aestivum*), and durum (*Triticum durum*). The life cycle of this fungal pathogen includes the formation of fungal sclerotial bodies in place of normal seeds [1]. The production by *C*. *purpurea* of toxic and hallucinogenic alkaloids including ergometrine, ergotamine tartrate, and lysergic acid diethylamide causes a disease known as ergotism upon consumption of grain products affected by *C*. *purpurea* [1,2]. While cases of humans affected by ergot are rare today, ergot alkaloid contamination represents a serious problem in agriculture since grazing cattle are highly susceptible. Therefore, ongoing surveillance and knowledge of baseline levels of ergot contamination in grain products destined for export is critical to support cereal producers and exporters, who are at economic risk from ergot contamination of their crops.

Since mycotoxins produced by *C*. *purpurea* are the source of concern related to ergot contamination, several methods have been developed for the detection and determination of ergot alkaloids in grains, grasses, feeds, and foods. These include planar solid phase extraction [3], enzyme-linked immunosorbent assay [4] and liquid chromatography-tandem mass spectrometry [5]. Alternatively, since ergot produces dark purple sclerotia that take the place of seed in an infected plant, harvested grain can be easily graded for ergot contamination by determining the proportion (weight basis) of sclerotia by visual inspection. While this is a simple, low-cost method, it can be time consuming and labor intensive and may miss small sclerotial bodies. Using this approach, and acknowledging that ergot is ubiquitous in the environment, tolerance levels have been set for ergot sclerotia in grain products that affect prices for both producers and exporters [2]. However, recent reports of Egyptian authorities shifting towards more stringent guidelines and regulations for ergot sclerotia in grain shipments [6] has emphasized the need for producers and exporters to be knowledgeable about the microorganisms associated with these commodities and for tolerance levels to be set and respected. This applies equally to pathogens besides ergot that are not as easily detected but could be a source of trade disputes or used as a criterion for novel grading standards.

Molecular diagnostic methods offer a means to detect and quantify microorganism (including pathogen, symbiont, and commensal) DNA on plant material pre- and post-harvest, with the potential to provide a “molecular grade” for a grain products based on the presence and/or abundance of particular microorganisms. Moreover, molecular-based approaches may be more accurate and sensitive depending on the sample size of plant material that is tested. Seed washing followed by deep sequencing of the chaperonin-60 (*cpn60*) molecular barcode [7–9] can provide a detailed picture of the bacterial and fungal microbiota of these environments, along with quantitative data that correlates to biological activity [10]. Alternatively, specific microorganism-targeted molecular diagnostics can be used to quantify bacterial or fungal taxonomic markers in these same environments [10].

In this work, we hypothesize that DNA from grain-associated ergot bodies of *Claviceps purpurea* is detectable using a simple seed wash and that quantitative molecular diagnostic assays are correlated with visual determinations of ergot contamination. We assessed the molecular-based detection of *Claviceps purpurea* through microbiota profiling and by specific quantitative diagnostics. We have provided a proof-of-principle for molecular grading by examining harvested grain products for the presence of *C*. *purpurea*. The results provide a framework for the development of molecular diagnostic tools to provide a “molecular grade” for ergot or other pathogens via the detection and quantification of target DNA from grain samples, with implications for trade in agricultural commodities and suitability of cereal grain for food and feed especially at the on-farm level.

## Materials and methods

### Grain sources

**Fig 1:**
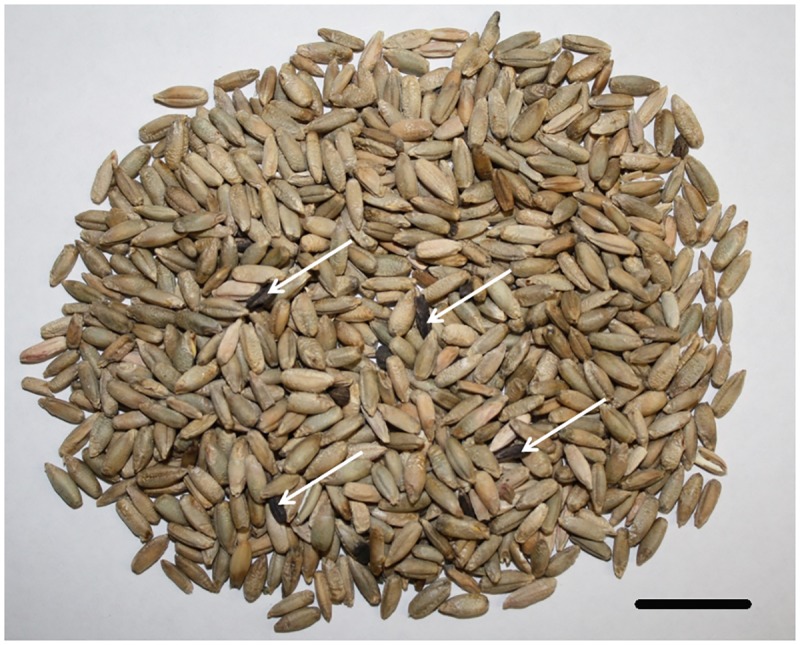
Ergot sclerotia observed in sample 9129 (Rye). Sclerotia are indicated by arrows. This sample had an ergot severity rating of 0.294% on a percentage weight basis (Table 2). Scale bar indicates 1 cm.

Grain samples were collected from the harvest sample program of the Canadian Grain Commission; therefore, no specific permissions were required. These studies did not involve any endangered or protected species. Ten samples of cereal grain from various locations that were downgraded for different factors (Table 1) were initially chosen for microbiome profiling using chaperonin-60 (*cpn60*) amplicon sequencing [11]. Subsequently, a series of 141 harvest samples from the 2014 crop year (Fig 1 and Table 2) that were rated for ergot contamination (weight/weight proportion of ergot sclerotia in the seed sample) were selected for detection and quantification of ergot DNA by the molecular diagnostic methods described below. Most grain samples were either wheat or durum, while a small number of non-cereal grain samples were also included. Samples were stored separately in plastic bags at room temperature before they were processed.

**Table 1:** Seed samples selected for microbiome profiling using *cpn60*.

Grain Type	Province^1^	Grain Class^2^	Variety	Grade	Grading factor^3^	Reads clustering with *C*. *purpurea cpn60* (LAMP result)	Total reads in dataset
Oats	NB	CEOats	Dieter	4CEOAT	FCLR	0 (neg)	17697
Wheat	ON	CEAD	Hallmark	CEFEED	FDK	0 (neg)	33540
Wheat	NS	CEHRW	-	3CEHRW	MIL	0 (neg)	31718
Wheat	ON	CERS	Sable	1CERS		1 (neg)	19410
Rye	QC	CERye	-	2CERYE	SPTD	0 (neg)	38379
Wheat	SK	CWRS	Pasqua	2CWRS organic	ERG	76 (pos)	38910
Wheat	SK	CWRS	Harvest	2CWRS organic	MDGE	34 (pos)	40873
Kamut	SK	Kamut	Khorasan	2KAMUT organic	MDGE	0 (neg)	48565
Canary seed	SK		Keet	Canary seed		42 (neg)	48691
Triticale	SK	Triticale	Tundel	3CWTriticale	ERG	2 (NT^4^)	37930

^1^NB, New Brunswick; ON, Ontario; NS, Nova Scotia; QC, Québec; SK, Saskatchewan

^2^CEOats, Canada Eastern Oats; CEAD, Canada Eastern Amber Durum wheat; CEHRW, Canada Eastern Hard Red Winter wheat; CERS, Canada Eastern Red Spring wheat; CERye, Canada Eastern Rye; CWRS, Canada Western Red Spring wheat

^3^FCLR, fair color; FDK, *Fusarium*-damaged kernels; MIL, mildew; SPTD, sprouted kernels; ERG, ergot; MDGE, midge damage

^4^NT, not tested

**Table 2:** Quantification of *C*. *purpurea* DNA in grain wash samples using ITS-targeted ddPCR and *cpn60*-targeted LAMP.

Sample	Province^1^	Description	Downgradingfactor	Ergotvalue (%)	ddPCR: ITSgenomes/g seed	LAMP: *cpn60* T_p_, minutes^2^	
						Calcein detection	Isothermal detection
7		Canadian brown mustard		0	78000	90.00	60.00
8		Canadian oriental mustard		0	5300	90.00	48.50
9		Canadian canola		0	3200	90.00	24.75
10		Canadian sample canola		0	4750	90.00	60.00
72	SK	Canadian Wheat	ergot	0.052	3895000	50.25	11.00
73	SK	Canadian Wheat	ergot	0.114	217000	53.75	11.00
201	MB	Buckwheat		0	15900	90.00	60.00
301	SK	Canadian Amber Durum	ergot	0.055	414500	61.00	15.00
414	SK	Canadian Wheat	ergot	0.05	301500	58.75	13.75
415	SK	Canadian Wheat	ergot	0.066	138000	76.25	18.25
481	SK	Canadian Wheat	ergot	0.053	105500	71.25	13.00
771	SK	Canadian Wheat	ergot	0.031	44500	68.50	11.00
865	AB	Canadian Amber Durum		0	32250	67.25	16.00
866	AB	Canadian Amber Durum		0	32300	63.00	17.00
927	AB	Canadian Wheat		0	24100	69.25	26.00
943	SK	Canadian Wheat	ergot	0.02	72500	64.75	19.25
1246	SK	Canadian Amber Durum	ergot	0.008	48450	64.00	15.25
1371	MB	Canadian Wheat	ergot	0.003	50400	64.00	12.50
1466	AB	Canadian Amber Durum		0	136000	78.50	24.50
1482	MB	Canadian Wheat	ergot	0.07	170500	61.25	13.25
1501	SK	Canadian Wheat	ergot	0.052	854500	49.00	11.75
1509	SK	Canadian Wheat	ergot	0.053	62000	63.75	14.25
1551	SK	Canadian Wheat	ergot	0.007	17950	69.25	24.50
1558	SK	Canadian Wheat	ergot	0.005	22250	65.75	18.25
1576	MB	Canadian Wheat	ergot	0.043	49750	69.50	14.25
1633	AB	Canadian Wheat		0	26800	72.25	18.50
1720	QC	Canadian Wheat	ergot	0.015	1001000	45.50	11.50
1865	SK	Canadian Wheat	ergot	0.008	31400	89.50	16.75
2054	AB	Canadian Wheat		0	24200	61.75	56.50
2075	AB	Canadian Wheat	ergot	0.018	77000	68.25	13.50
2340	SK	Canadian Triticale		0	15050	66.75	14.25
2352	MB	Canadian Wheat		0	9250	73.75	56.25
2387	AB	Canadian Amber Durum	ergot	0.031	176500	69.00	21.75
2389	AB	Canadian Amber Durum	ergot	0.049	122500	59.00	12.00
2472	SK	Canadian Amber Durum	ergot	0.014	71000	66.25	15.00
2522	AB	Canadian Wheat	ergot	0.052	880000	55.00	11.75
2598	MB	Canadian Wheat		0	2350	64.25	22.25
2599	SK	Canadian Wheat		0	8250	90.00	3.00
2640	MB	Canadian Wheat	ergot	0.022	479500	46.50	11.00
2875	MB	Canadian Wheat	ergot	0.015	87000	47.00	60.00
3040	SK	Canadian Amber Durum		0	507000	54.00	16.00
3062	QC	Canadian Wheat	ergot	0.12	154500	51.00	14.75
3119	SK	Canadian Amber Durum	ergot	0.037	74500	67.50	14.25
3195	AB	Canadian Wheat	ergot	0.06	253000	54.25	11.75
3513	MB	Canadian Rye	ergot	0.01	51450	66.50	13.50
3662	MB	Canadian Wheat	ergot	0.058	192000	74.25	17.75
3700	AB	Canadian Wheat	ergot	0.086	700000	47.50	10.75
3843	SK	Canadian Amber Durum	ergot	0.045	134500	67.75	18.00
3856	AB	Canadian Wheat	ergot	0.063	6745000	52.00	12.50
3889	QC	Canadian Wheat		0	4700	90.00	20.75
3949	MB	Canadian Wheat	ergot	0.051	132000	70.25	13.25
4070	SK	Canadian Rye	ergot	0.12	421000	54.00	11.25
4179	AB	Canadian Wheat	ergot	0.067	19100000	51.00	14.50
4182	AB	Canadian Wheat	ergot	0.076	551000	55.25	15.50
4442	AB	Canadian Triticale	ergot	0.1	87450	48.00	28.50
4535	MB	Canadian Wheat	ergot	0.079	33550	68.75	21.50
4580	BC	Canadian Wheat		0	27800	51.25	14.50
4600	SK	Canadian Wheat		0	440500	50.75	16.00
4637	MB	Canadian Wheat	ergot	0.016	26800	68.25	12.75
4696	SK	Canadian Wheat		0	8850	62.25	13.75
4739	SK	Canadian Wheat	ergot	0.058	1425000	50.00	8.50
4740	SK	Canadian Amber Durum	ergot	0.011	76050	72.25	21.25
4741	SK	Canadian Wheat	ergot	0.071	413500	56.25	17.25
4757	SK	Canadian Amber Durum	ergot	0.01	27450	73.50	19.25
4759	SK	Canadian Wheat	ergot	0.037	1664500	45.75	43.00
4780	AB	Canadian Wheat		0	387500	66.25	11.75
4860	SK	Canadian Rye	ergot	0.022	1883000	49.00	12.25
4947	AB	Canadian Wheat		0	181500	53.25	13.50
5005	AB	Canadian Amber Durum	ergot	0.064	1682500	56.00	13.00
5134	SK	Canadian Amber Durum	ergot	0.187	3560000	54.25	13.00
5154	SK	Canadian Wheat	ergot	0.006	14550	89.50	21.00
5162	AB	Canadian Wheat		0	14700	90.00	29.00
5175	AB	Canadian Wheat		0	23100	62.75	16.75
5185	SK	Canary Seed		0	839500	71.50	13.50
5202	SK	Oat Spelt		0	27500	75.00	14.00
5289	MB	Canadian Wheat	ergot	0.003	6550	90.00	19.50
5447	SK	Canadian Amber Durum	ergot	0.219	1635000	56.25	12.75
5545	SK	Canadian Amber Durum	ergot	0.015	68950	55.25	15.25
5569	MB	Canadian Wheat	ergot	0.036	237000	60.75	11.75
5570	SK	Canadian Wheat	ergot	0.024	245000	62.75	15.75
5573	SK	Canadian Wheat	ergot	0.019	122000	65.25	14.00
5599	SK	Canadian Wheat		0	15950	58.75	20.50
5610	SK	Canadian Amber Durum	ergot	0.01	252500	50.50	15.50
5650	AB	Canadian Wheat	ergot	0.04	5645000	38.75	9.25
6061	SK	Canadian Amber Durum	ergot	0.015	33650	70.00	45.00
6103	MB	Canadian Wheat	ergot	0.019	38250	76.25	21.25
6117	AB	Canadian Amber Durum	ergot	0.02	127000	45.50	11.75
6232	MB	Canadian Rye	ergot	0.05	220500	53.75	13.00
6629	AB	Canadian Amber Durum	ergot	0.064	239000	60.50	14.75
6907	MB	Canadian Wheat	ergot	0.071	82000	61.50	19.50
7026	SK	Canadian Wheat	ergot	0.128	751500	47.75	12.50
7352	SK	Canadian Rye	ergot	0.05	605000	54.50	16.75
7458	SK	Canadian Wheat	ergot	0.08	8300000	40.50	11.75
7519	SK	Canadian Wheat	ergot	0.047	2845000	39.00	9.75
7686	SK	Canadian Amber Durum	ergot	0.016	59200	54.50	13.75
7758	AB	Canadian Wheat	ergot	0.067	145000	65.75	15.25
7777	SK	Canadian Amber Durum	ergot	0.091	3035000	44.50	12.25
7864	AB	Canadian Wheat	ergot	0.1	247500	53.00	10.75
7866	AB	Canadian Wheat	ergot	0.22	1336500	43.05	11.50
7950	SK	Canadian Wheat	ergot	0.087	4295000	52.25	13.00
8006	SK	Canadian Wheat		0	9250	90.00	11.00
8094	MB	Canadian Rye		0	7145000	52.25	60.00
8110	SK	Canadian Wheat	ergot	0.05	13550	64.25	17.25
8292	SK	Canadian Amber Durum	ergot	0.042	301500	62.75	13.25
8393	MB	Canadian Wheat	ergot	0.058	2225000	49.50	29.75
8394	SK	Canadian Amber Durum	ergot	0.031	12050	59.00	12.25
8408	AB	Canadian Wheat	ergot	0.012	42500	66.50	11.25
8549	SK	Canadian Rye	ergot	0.064	3175000	50.50	13.50
8597	SK	Canadian Amber Durum		0	106000	67.00	22.50
8671	SK	Canadian Wheat	ergot	0.056	46700	80.50	20.25
8703	SK	Canadian Amber Durum	ergot	0.044	104500	55.75	14.75
8715	AB	Canadian Wheat	ergot	0.023	89000	59.50	17.50
8719	AB	Canadian Wheat	ergot	0.006	25250	67.25	13.50
8803	SK	Canadian Wheat		0	39450	79.75	16.50
8859	SK	Canadian Wheat		0	85000	58.75	14.25
8864	MB	Canadian Wheat	ergot	0.085	1656000	50.50	16.00
8866	MB	Canadian Rye		0	41000	53.25	19.25
8929	AB	Canadian Wheat		0	6900	90.00	19.25
8977	SK	Canadian Wheat		0	14250	90.00	17.50
9064	AB	Canadian Wheat	ergot	0.08	818000	47.25	12.25
9067	MB	Canadian Wheat		0	9400	90.00	20.25
9068	AB	Canadian Wheat	ergot	0.1	8650000	49.25	17.50
9076	SK	Canadian Wheat	ergot	0.084	410500	48.00	12.50
9101	MB	Canadian Wheat	ergot	0.05	184000	62.00	17.25
9106	SK	Canadian Wheat	ergot	0.013	133500	61.75	12.00
9112	SK	Canadian Wheat	ergot	0.026	1876000	62.25	15.50
9121	AB	Canadian Wheat	ergot	0.069	13850	75.50	14.75
9122	AB	Canary Seed		0	30050	61.50	15.00
9129	MB	Canadian Rye	ergot	0.294	5865000	89.50	13.00
9154	AB	Canadian Wheat	ergot	0.007	24400	59.50	14.25
9213	AB	Canadian Wheat	ergot	0.067	743000	73.25	12.75
9411	ON	Canadian Wheat		0	114000	48.75	18.25
9420	AB	Canadian Rye		0	92500	63.75	13.25
9426	QC	Canadian Wheat		0	3050	79.25	47.50
9440	AB	Canadian Wheat	ergot	0.066	17300	55.00	13.50
9459	MB	Canadian Wheat	ergot	0.007	148500	54.00	19.50
9484	MB	Canadian Wheat	ergot	0.003	24450	67.75	26.25
9658	SK	Canadian Amber Durum	ergot	0.02	106500	55.50	16.00
9952	SK	Canadian Wheat	ergot	0.05	178500	90.00	12.75
9968	AB	Canadian Wheat	ergot	0.061	191000	90.00	18.50
9985	ON	Canadian Wheat		0	30700	90.00	20.75

^1^AB, Alberta; BC, British Columbia; MB, Manitoba; ON, Ontario; QC, Québec; SK, Saskatchewan

^2^Samples assayed by LAMP were given a T_p_ of 90 (calcein detection) or 60 (isothermal detection) if no signal was observed during the assay

### DNA extraction from grain-associated epiphytic microbiota and profiling the microbial communities

DNA was extracted from grain washes as described [10]. Briefly, 25 g subsamples of grain were soaked in 45 ml of buffered peptone water containing 0.05% Triton X-100 (Sigma, St. Louis, MO) in a 250 ml Erlenmeyer flask at room temperature with shaking (150 rpm) for 1 hour. The liquid fractions were centrifuged at 4000 × *g* for 15 minutes and the supernatant discarded. Pellets were resuspended in 200 μl of TE buffer and subjected to DNA extraction using a previously described bead-beating protocol [12]. DNA was quantified using a Quant-IT DNA quantification kit and Qubit fluorometer (Invitrogen, Burlington, Ontario). To account for the possible presence of PCR inhibitors in the extract, a dilution series of one of the grain wash samples was prepared and each dilution used as a template for *cpn60* universal PCR as described [10]. The dilution that provided the strongest bands upon agarose gel electrophoresis (1:5) was used as template for *cpn60* amplicon generation and sequencing. Purified, concentrated amplicons from all grain samples were pooled on an equimolar basis prior to emPCR adaptor ligation and pyrosequencing using Titanium chemistry (Roche) as described previously [8,13]. For quantification of ergot in grain using molecular diagnostic assays, DNA was prepared in a similar manner except that 10 g of grain were used and the final solution was further purified using Agencourt AMPure XP beads (Beckman-Coulter) at 1:1 (v/v) bead:DNA solution according to the manufacturer’s recommendations. DNA samples were eluted from the beads with 30 μl of 10 mM Tris-Cl pH 8.0.

### Determination of taxonomic marker gene sequences from *C*. *purpurea* and ergot sclerotia

Sclerotia of ergot (Table 3) were obtained from field-grown samples from Manitoba, Canada. Sclerotia were crushed in liquid nitrogen using a mortar and pestle and the powder was subjected to a bead beating protocol as previously described [12], or using a DNeasy Plant DNA mini kit (Qiagen). The final volume of DNA solution was 200 μl. The DNA was used as template for amplification of the fungal internal transcribed spacer (ITS) fungal barcode using primers ITS4/ITS5 as previously described [14]. PCR products of ~625 bp were cloned into pGEM-T Easy (Promega) according to the manufacturer’s recommendations, and individual colonies were selected from each sample for sequencing. To amplify the Cpn60-encoding gene [15], PCR primers targeting fungal *cpn60* sequences (S1 Table) were used to generate PCR products from sample erg0256 (Table 3). PCR for *cpn60* used 1x *Taq* buffer (Invitrogen); 2.5 mM MgCl_2_; 500 nM each dNTP; 400 nM each primer; and 1 U *Taq* DNA polymerase (Invitrogen). The sequences of these individual amplicons spanned regions upstream and downstream of the *cpn60* universal target (*cpn60* UT) [15], but contained a gap. To determine the complete sequence of the *C*. *purpurea cpn60* UT, primers based on these sequences were designed to amplify a product of 741 bp that contained the entire *cpn60* UT (S1 Table; concentrations of all components in PCR as described above). These 741 bp amplicons were generated from all samples and were cloned into pGEM-T Easy. After sequence determination from individual clones, the 555-bp UT region was manually extracted and used for analysis. To determine the reference *cpn60* and ITS sequences of *C*. *purpurea*, reference strain 714 was obtained from Deutsche Sammlung von Mikroorganismen und Zellkulturen (DSMZ). The organism was cultured on 10 cm petri dishes containing YpSs medium (4 g/L yeast extract, 15 g/L soluble starch, 0.5 g/L MgSO_4_ · 7 H_2_O, 1.0 g/L KH_2_PO4, and 15 g/L agar) overlayed with a sterile 0.1 μm polycarbonate membrane filter (Sterlitech). After 14 d at 25°C, a colony of approximately 2 cm diameter was retrieved from the plates using sterile forceps and crushed in liquid nitrogen. Aliquots of the powder (~100 mg each) were used for DNA extraction with a DNeasy Plant Mini kit (Qiagen). DNA so obtained was used as a template for PCR with ITS primers [14] and with primers D0282/D0283 (S1 Table) to determine the reference ITS and *cpn60* UT sequences, respectively.

**Table 3:** ITS and *cpn60* clone diversity observed in sclerotia sourced from Manitoba, Canada.

Sample	Ergot rating (%)	*cpn60* clones (UT region)				ITS clones			
		Number of clones examined	Number of distinct sequences	Percent identity to one another	Length (base pairs)	Number of clones examined	Number of distinct sequences	Percent identity to one another	Length (base pairs)
erg0252	0.02	18	2	99–100	555	21	3	98–100	625–628
erg0253	0.023	21	8	98–100	555	23	4	99–100	623–625
erg0254	0.012	20	16	98–100	555	19	10	98–100	623–625
erg0256	0.05	24	12	98–100	555	22	8	99–100	622–625
erg0258	0.023	23	15	98–100	555	21	10	99–100	622–624
Total number of clones examined			106				106		
Total number of unique sequences			53				35		

### Quantitative PCR for *C*. *purpurea* based on ITS sequences

A representative ITS sequence obtained from an ergot sclerotium (erg0256; see Table 3) was used as a basis for hydrolysis probe assay design using Beacon Designer v.7.90 (Premier Biosoft, Palo Alto, CA) (S1 Table). Amplification primers and the hydrolysis probe were purchased from Integrated DNA Technologies (Coralville, IA). To determine the PCR efficiency, a set of standards was prepared using ITS-containing plasmid DNA. DNA was prepared using a miniprep kit (Qiagen), and the DNA was linearized using *Pst*I. The concentration of linearized plasmid DNA was determined in triplicate using a Qubit instrument (BR kit, Life Technologies). The mean concentration (ng/μl) was converted to copies/μl using an approximate molecular weight of 650 g/mol per base pair. This solution was diluted to provide 10^7^–10^1^ copies per assay and used as control templates in qPCR. Reactions used SsoFast Universal probes supermix (Bio-Rad, Mississauga, ON, Canada) in a 20 μl final volume with 300 nM of each primer and 200 nM of probe. Amplification was carried out using a CFX96 real-time system with a C1000 base (Bio-Rad) and reactions were quantified using BioRad CFX manager software (v.3.1). The slope of the line resulting from plotting threshold cycle (C_q_) values vs. log_10_ copy number was used to determine PCR efficiency according to E = 10^(-1/slope)^, where 2.0 is theoretical [16]. To obtain quantification results that were independent of standards, the assay was adapted to the droplet digital PCR (ddPCR) format. Reaction conditions for ddPCR were first optimized using gradient PCR (54–65°C). Reactions used ddPCR supermix for probes (Bio-Rad) and had 900 nM of each primer and 250 nM of hydrolysis probe in a 20 μl reaction volume. The accuracy of ddPCR quantification was examined using a dilution series of known copy numbers (standard curve prepared as described above). To quantify *C*. *purpurea* in intact grain wash extracts, template DNA prepared as described above was digested using *Eco*RI (37°C, 60 min, then 85°C, 5 min) and 2 μl of the digested DNA was added to the ddPCR mixture. Emulsions were prepared prior to amplification using a QX100 droplet generator (Bio-Rad), and amplifications were done using a C1000 Touch thermocyler (BioRad). After amplification, positive and negative droplets were quantified using a QX100 droplet reader (Bio-Rad) and the proportion of negative droplets was converted to copies per well using QuantaSoft v.1.6.6 (Bio-Rad). Results reported by QuantaSoft were converted to copy number/g grain extracted by correcting for sample preparation. In cases where very high counts were observed, samples were diluted accordingly.

### Detection of *C*. *purpurea* DNA in seed washes using Loop-Mediated Isothermal DNA Amplification (LAMP) based on *cpn60*

Amplification primers for LAMP (S1 Table) that targeted *cpn60* of *C*. *purpurea* were designed using LAMP Designer v. 1.12 (Premier Biosoft, Palo Alto, CA). LAMP conditions were as described for detection using calcein [17]. The same primers were also used for amplification and detection using Isothermal Mastermix (Prolab Diagnostics, Richmond Hill, ON, Canada), which features proprietary detection chemistry that also facilitates the determination of product annealing temperature. For both detection chemistries, a temperature of 63°C was used for amplification. Reactions were monitored in real time using a Genie II or Genie III instrument (OptiGene, Horsham, UK) and the time to positive (T_p_) was reported by the instrument.

### Assay parameters

The performance characteristics of the molecular diagnostic assays were determined according to established standards [18,19]. Analytical specificity was examined by using DNA isolated from a panel of fungi that grow in association with one or more of the field crops typically grown in Canada, including *Alternaria* spp, *Fusarium* spp., *Stemphylium* sp., *Rhizoctonia* sp., *Plectosphaerella* sp., *Leptosphaeria* spp., and *Verticillium* sp. To determine the limit of detection (LOD) of the LAMP assay, a series of 6 serial dilutions of *C*. *purpurea* DNA was added to grain wash DNA that was determined to lack detectable ergot DNA and a total of 70 replicates were analyzed using probit (SPSS). The weight of DNA (ng) added to each assay was converted to genome equivalents using a genome size of 32.1 Mbp [20] and 650 g/mol per base pair. The LOD was defined as the number of *C*. *purpurea* genome equivalents that yielded positive results 95% of the time [18]. The linearity of the LAMP assay was examined by polynomial regression analysis of the T_p_ determined over a wider range of dilutions and including 3–14 replicates in each dilution. Intra-assay precision was determined by calculating the coefficient of variation of the T_p_ determined at each of three levels (near the LOD, at twice the LOD, and at 20 times the LOD). Finally, assay sensitivity and specificity were determined by scoring the numbers of positive and negative results obtained using visual inspection as a gold standard and calculating these parameters as described [19].

## Results

### Microbial community profiling of downgraded grain

The epiphytic microbiota of grain that had been downgraded for various factors including *Fusarium*, mildew, ergot, and midge damage [11] were profiled. DNA extracts representing seed epiphytic microorganisms were characterized by sequencing PCR amplicons using pyrosequencing. A total of 355715 reads produced 3609 assembled, unique *cpn60* UT sequences (operational taxonomic units, OTU) after processing with mPUMA [21]. In addition to the bacterial OTU that were observed, sequences similar to Sordariomycetes such as *Cylindrocarpon*, *Magnaporthe*, *Fusarium*, and *Verticillium* spp were observed but they had relatively low sequence identities to known strains (~85%). Two OTU in particular, OTU02794 and OTU03634, clustered with fungal sequences from cpnDB but appeared to occupy a gap in the reference database. These fungal OTU were found at low levels in several of the datasets (Table 1), but they could not be further identified due to the lack of reference data.

### Identification of fungal OTU as *C*. *purpurea*

**Fig 2:**
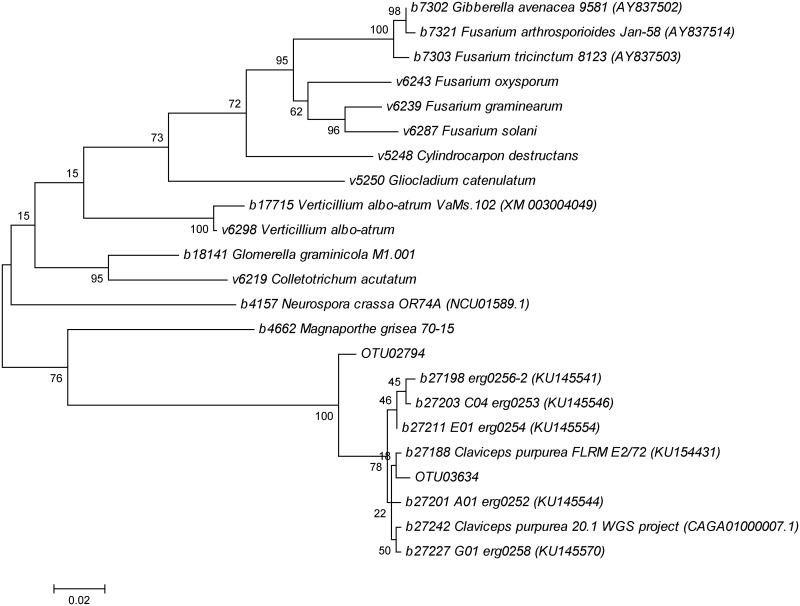
Phylogenetic analysis of *cpn60* UT sequences derived from microbial profiling and ergot sclerotia compared to reference sequences. Sequences are prefixed by cpnDB ID number (www.cpndb.ca) and GenBank accession numbers (www.ncbi.nlm.nih.gov) are provided in parentheses where available. The tree was calculated using the Maximum Likelihood method based on the Tamura-Nei model [22] using MEGA6 [23]. The tree was bootstrapped (100 iterations) and numbers next to the nodes indicate the percentage of trees in which the associated taxa clustered together. Branch lengths correspond to the number of substitutions per site.

BLAST analysis showed that the ITS sequences that were determined from 5 ergot sclerotia (Table 3) were closely related to previously reported ITS sequences from *C*. *purpurea*, with > 99% identity observed (data not shown). This suggested that these sclerotia consisted primarily of *C*. *purpurea* DNA, but as these were environmental samples it was assumed that DNA from other organisms was also present. Examining the *cpn60* sequences generated from these sclerotia revealed that these sequences clustered closely with the sequence obtained from the reference strain of *C*. *purpurea* (DSMZ 714), and with a *cpn60* sequence that was extracted from a genome sequence of *C*. *purpurea* [20] (Fig 2). Thus, the *cpn60* sequences determined from the ergot sclerotia indeed corresponded to the sequence of *C*. *purpurea cpn60*. In addition, the previously unidentified OTU from the microbiome profiling of the downgraded grain lots were thereby identified as deriving from *C*. *purpurea* (Fig 2).

### Ergot sclerotia contain many copies of *cpn60* and ITS

Each of the five ergot sclerotia contained 3–10 distinct copies of ITS (Table 3), all of which had high sequence identities with previously reported ITS sequences for *C*. *purpurea* [24]. The *cpn60* sequences revealed an even higher heterogeneity in most samples, with 2–16 distinct sequences observed (Table 3, S1 Fig). Like the ITS sequences, the sclerotia *cpn60* sequences were closely related to sequences determined from the *C*. *purpurea* reference strain (DSMZ 714) and the *C*. *purpurea* genome. To determine the likely copy number of *cpn60* and ITS within the *C*. *purpurea* genome, we used representative sequences from ergot sclerotia to query the genome sequence by BLAST. Using a scelerotium-derived ITS sequence as query for blastn, only a single hit was observed in the *C*. *purpurea* genome (GenBank CAGA00000000.1), with an e-value set at 1000 (data not shown). Similarly, using tblastx, only a single match was observed in the *C*. *purpurea* genome using the translated amino acid sequence of *cpn60* as query (data not shown).

### Quantitative molecular diagnostic assays of grain washes were correlated with visual assessments of ergot load

**Fig 3:**
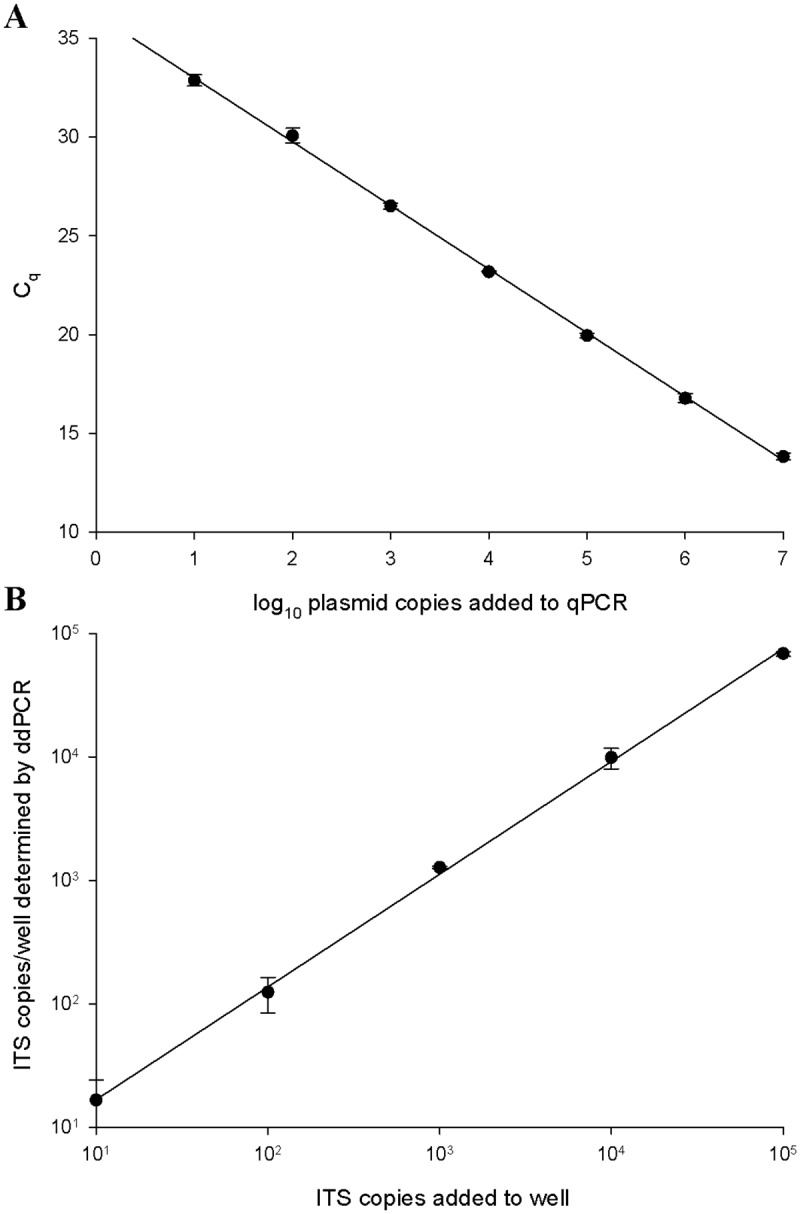
ITS-targeted qPCR assay linearity assessed by standard curve (A) or ddPCR calibration curve (B).

The qPCR assay that was designed to detect the ITS sequence of *C*. *purpurea* was highly efficient (E = 2.04; r^2^>0.999), and detected as few as 10 copies of target DNA per reaction (Fig 3A). Similarly, the ddPCR-adapted version of the assay was highly accurate; the number of copies added to each assay was reported correctly at levels of input ITS copy numbers ranging from 10 to 10^5^ copies (Fig 3B).

The ITS-targeted ddPCR assay was positive with all grain wash samples, including 37 unrated/negative samples and four samples from canola, which is not a host for *C*. *purpurea* (Table 2). Although the ddPCR assay did not yield a signal with the reference fungi used to determine analytical specificity, this observation suggested that the assay may generate a signal with nontarget grain-associated microorganisms. Nevertheless, a positive, highly significant correlation was observed between the number of *C*. *purpurea* genomes detected by ddPCR and ergot severity of a percentage weight basis (Table 4).

**Table 4:** Spearman rank correlation between ergot severity (% weight basis) and molecular quantification of ergot DNA in grain wash templates.

method	target	unit	Spearman correlation (ρ)	p-value	n
ddPCR	ITS	*C*. *purpurea* genomes/g	0.636	2.0x10^-7^	141
LAMP(calcein)	*cpn60*	T_p_, minutes	-0.449	3.1x10^-8^	141
LAMP (isothermal)	*cpn60*	T_p_, minutes	-0.423	2.2x10^-7^	141

**Fig 4:**
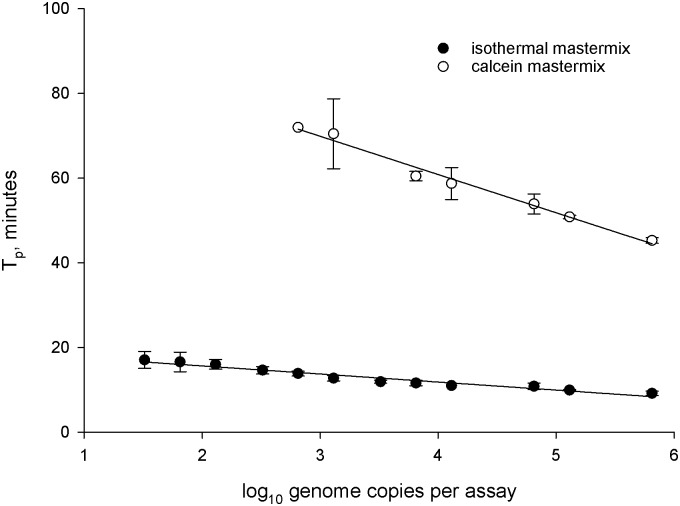
*cpn60*-targeted LAMP assay linearity assessed by expressing T_p_ related to *C*. *purpurea* genome copies using the two LAMP detection systems evaluated in this study. The equations for each curve are: y = -9.03x+96.98 (calcein detection) and y = -1.90x+19.44 (isothermal detection). The correlation coefficients (r^2^) are 0.99 (calcein detection) and 0.95 (isothermal detection).

The *cpn60*-targeted LAMP assay in both detection formats was apparently specific, as none of the fungal isolates examined provided evidence of cross-reactivity (data not shown). In addition, the samples that had been profiled by *cpn60* sequencing were examined using LAMP and none of the samples that lacked *C*. *purpurea* reads were positive in the LAMP assay (Table 1). The LOD of the LAMP assay with Isothermal detection chemistry was approximately 75 genome equivalents. The T_p_ of the assay in both formats showed a strong, inverse correlation to input template amount, and the Isothermal detection format was positive in less than 10 minutes at higher template amounts (Fig 4). The slopes of the two detection chemistries were quite different, with calcein detection featuring a steeper curve and much slower detection compared to the Isothermal detection chemistry (Fig 4). The LAMP assay also featured a reasonable intra-assay variability at the three input levels examined (Table 5).

**Table 5:** Intra-assay reproducibility of the *cpn60*-targeted LAMP (isothermal detection).

level	dilution	genomes/assay	CV^1^	n
high	500	1298.35	0.052	3
low	5000	129.83	0.072	3
LOD	10000	64.92	0.117	10

^1^CV, coefficient of variation; standard deviation/mean

Unlike the ITS-targeted ddPCR assay, the LAMP assay targeting *cpn60* was not positive in all of the grain wash samples analyzed. With calcein detection, 16 of the 141 samples, including the four canola grain wash samples, tested negative (Table 2). However, using the apparently more sensitive Isothermal detection format (Fig 4), only 5 samples (canola -7; canola-10; buckwheat-201; wheat-2875; and rye-8094) tested negative. To determine if samples providing discordant results contained amplifiable *C*. *purpurea* DNA, the calcein-negative grain wash templates were amplified using primers D0282-D0283 (S1 Table). Amplicons were generated for samples canola-9, canola-10, wheat-9952, and wheat-9985. The sequences of the amplicons indicated that the wheat samples indeed contained *C*. *purpurea* genomic DNA, while the canola samples yielded amplicons that did not correspond to *C*. *purpurea* (data not shown). These observations indicate that the LAMP assay in both formats, as well as the PCR assay, can generate false positive and false negative results, although in the case of the Isothermal detection format the false positive results were very late and could be avoided by reducing the assay time.

Despite this, the LAMP assay (calcein detection) generated quantitative data that correlated inversely with ergot severity, with a statistically significant *p*-value (Table 4). Moreover, the sensitivity of the LAMP assay using visual rating as a gold standard was very high (0.97 using calcein detection or 0.99 using Isothermal detection), with only 3/100 (calcein) or 1/100 (Isothermal) positive samples generating a negative result with LAMP (Table 6). This is consistent with a low false negative rate, or type I error [19]. Conversely, the specificity of the LAMP assay was apparently low in both formats, suggesting a high false positive rate (type II error). With Isothermal detection, more of the grain wash samples tested positive, consistent with the increased analytical sensitivity of the assay in this format (Fig 4). Like the calcein detection format, the T_p_ of the LAMP assay with Isothermal detection chemistry was inversely, significantly correlated to ergot levels determined using visual inspection (Table 4).

**Table 6:** Sensitivity and specificity of the *C*. *purpurea cpn60*-targeted LAMP assay compared to visual rating (gold standard).

	test: visual examination		
test: *cpn60*-targeted LAMP	positive	negative	total
positive	97	28	125
negative	3	13	16
total	10	41	141
		95% confidence interval	
test sensitivity	0.97	0.033	
test specificity	0.32	0.142	

## Discussion

*Claviceps purpurea* is a pathogen of grasses and cereals that co-evolved with its host in the Cretaceous period, at least 100 million years ago [25]. The pathogen is therefore expected to be ubiquitous in the environment; this fact, combined with the danger posed to humans and animals associated with the consumption of ergot alkaloids, makes ongoing surveillance necessary to protect cereal grain and end products from ergot contamination. While laboratory-based methods can quantify ergot alkaloids effectively, the level of ergot contamination in harvested grain can be determined visually by picking and weighing sclerotia from representative subsamples (e.g. Fig 1) of most cereals. One exception to this is canary seed, which is not typically rated due to seed size, but is also not normally considered for human consumption. In Canada, canary seed is not considered an official grain, and the industry is free to establish its own quality criteria. However, a recently registered hairless variety of canary seed is intended for human consumption (http://www.hc-sc.gc.ca/fn-an/gmf-agm/appro/canary-seed-lang-graine-alpiste-decision-eng.php) [26], so it is expected that there will be some need for ergot determination on canary seed in the near future. This would likely be done on ground material rather than grain washes.

The detection of ergot and other pathogen DNA in grain wash samples can be readily accomplished using sequencing methods, which can provide a profile of the bacterial and fungal microbiome associated with the grain lot under analysis [10]. This method has the major advantage of being non-targeted; rather than querying a sample for the presence of a particular pathogen, microbiome profiling can determine if the sample under analysis contains DNA from any potential pathogen of concern. However, despite all of the improvements in sequencing technology, screening hundreds of samples in this way would still be a difficult and rather expensive undertaking. Moreover, the results can be somewhat ambiguous. For example, a nucleotide sequence identity or read abundance cutoff may need to be established for various pathogens to determine an actionable quarantine pathogen detection threshold. The consequences of such decisions can be important, especially regarding grade, end use markets and trade measures. Finally, as we have shown, the success of a microbiome profiling method for detecting pathogen DNA is limited by the breadth of the reference database, since a sequence can only be identified by comparison to known reference sequences.

We determined the microbial profile of a range of cereal grain samples that had been downgraded for various reasons, including ergot contamination. Reads from sequencing datasets that were initially unidentified were found to correspond to *C*. *purpurea cpn60*, which emphasizes the importance of continuously enhancing reference databases. Despite the fact that reads corresponding to *C*. *purpurea* were detected in the samples profiled using *cpn60* universal PCR, purified DNA from ergot sclerotia as well as from the reference strain of *C*. *purpurea* failed to generate a *cpn60* amplicon using these same primers; a modified set of universal PCR primers targeted to fungal *cpn60* was required for this purpose (S1 Table). These observations are consistent with our previous work with *Alternaria* and *Fusarium* [10], and may be explained in part by the difference in primer/template ratios between complex template and single-template PCR. Nevertheless, we successfully amplified and sequenced *cpn60* and ITS from ergot sclerotia, and the detection of multiple related copies of both genes suggests that a single sclerotium contained a cluster of related strains of *C*. *purpurea* rather than a single strain. This is supported by the fact that a single copy of both genes was found in the genome of *C*. *purpurea*.

We have investigated the feasibility of applying targeted molecular diagnostic assays to the detection of *C*. *purpurea* DNA from grain samples. We used unground grain samples in the current study, which leaves ergot sclerotia intact. Despite this, we were able to detect *C*. *purpurea* DNA in grain washes of samples that were known to be contaminated with ergot. It is unknown if grinding the seed samples, which may release more *C*. *purpurea* DNA from contaminating sclerotia but would also release large quantities of host DNA and potentially interfering starch, which may have facilitated or hampered the detection. Nevertheless, the ITS-targeted ddPCR assay generated quantitative results that were highly correlated to ergot severity as determined by visual rating, but all samples tested positive. This made the calculation of assay sensitivity and specificity compared to visual rating impossible and suggests that the ddPCR assay we described suffered from a low analytical specificity, which is observed when nontarget analytes generate a signal [18]. In contrast, the *cpn60*-targeted LAMP assay was apparently discriminatory for *C*. *purpurea* DNA, since neither the nontarget genomic DNA templates nor the *C*. *purpurea*-negative samples profiled by sequencing (Table 1) generated a signal. This is not surprising, since LAMP is thought generally to feature a higher analytical specificity than PCR [17]. The LAMP assay is also rapid, inexpensive, and adaptable to use outside of the laboratory environment; these advantages make it analogous to visual-based rating. Moreover, the assay we have described generated quantitative data that correlated strongly and inversely with ergot severity, irrespective of the detection chemistry used.

The *cpn60*-LAMP assay featured a very low false negative rate compared to visual rating; in other words, virtually all samples in which ergot sclerotia were visually observed tested positive. The apparently high false positive rate for LAMP could be attributed to the fact that some samples like canary seed were not rated but given a score of 0 for ergot. Alternatively, this could be attributed to molecular detection being more sensitive than visual inspection. In addition, the ubiquity of *Claviceps* spores or sclerotia and their dispersion through wind, insects, and mechanical means [27], combined with the relative stability of DNA, could lead to target detection in the absence of observable disease. However, the *Brassica napus* seed lots we investigated tested negative for ergot by the *cpn60*-targeted LAMP (calcein detection), suggesting that *C*. *purpurea* DNA is not detected on the seeds of plants that are non-hosts. It is possible that *C*. *purpurea* DNA could be detected on the seeds of other canola samples, since cross-contamination between infected cereals and canola seeds could occur during growth or transport.

The rejection in 2016 of grain imports by Egypt, the world’s largest wheat importer, has clearly demonstrated that importing countries can arbitrarily set standards for any pathogen that have a major socio-economic impact. This suggests that an understanding of the microorganisms associated with agricultural commodities is extremely important for producers, exporters, and importers. Microbial community profiling is one way to approach this, but it has limitations. Pathogen-specific diagnostics can be used to overcome some of these, but they are only capable of querying one or a few organism(s) at a time. In this work, we have chosen ergot to demonstrate these points because it was initially unidentifiable in the sequencing datasets, there is a simple, quantitative visual diagnostic available, and there are recent, serious trade issues associated with ergot contamination. However, not all plant diseases that could contaminate grain can be identified visually or are present externally. The molecular diagnostic methods we have described for the detection of ergot could easily be adapted to other target pathogens.

## Supporting information

S1 Table Primer/Probe sequences and amplification conditions. (XLSX)

S2 Table Distance matrices of the sequences examined from individual sclerotia and reference sequences. (XLSX)

S1 Fig Phylogenetic analysis of *cpn60* and ITS sequences amplified from individual sclerotia (Table 3). (PPTX)

S1 File *cpn60* nucleotide sequences determined from all sclerotia examined (Table 3). (TXT)

S2 File Cpn60 peptide sequences determined from all sclerotia examined (Table 3). (TXT)

S3 File ITS nucleotide sequences determined from all sclerotia examined (Table 3). (TXT)

## References

[1] Kendrick B (1992) The Fifth Kingdom. Newburyport, MA: Focus Information Group.

[2] Tittlemier SA, Drul D, Roscoe M, McKendry T (2015) Occurrence of ergot and ergot alkaloids in Western Canadian wheat and other cereals. Journal of Agriculture and Food Chemistry 63: 6644–6650. 10.1021/acs.jafc.5b02977 26134095

[3] Oellig C, Melde T (2016) Screening for total ergot alkaloids in rye flour by planar solid phase extraction–fluorescence detection and mass spectrometry. Journal of Chromatography A 1441: 126–133. 10.1016/j.chroma.2016.02.075 26947163

[4] Flieger M, Wurst M, Shelby R (1997) Ergot alkaloids—Sources, structures and analytical methods. Folia Microbiologica 42: 3–30. 9160999 10.1007/BF02898641

[5] Guo Q, Shao B, Du Z, Zhang J (2016) Simultaneous Determination of 25 Ergot Alkaloids in Cereal Samples by Ultraperformance Liquid Chromatography-Tandem Mass Spectrometry. J Agric Food Chem 64: 7033–7039. 10.1021/acs.jafc.6b02484 27584949

[6] Dahan ME, Knecht E (2016) TIMELINE-Egypt's on again, off again relationship with ergot. Reuters: Reuters.

[7] Tian Q, Zhao W, Lu S, Zhu S, Li S (2016) DNA Barcoding for Efficient Species- and Pathovar-Level Identification of the Quarantine Plant Pathogen Xanthomonas. PLoS One 11: e0165995 10.1371/journal.pone.0165995 27861494 PMC5115671

[8] Schellenberg J, Links MG, Hill JE, Hemmingsen SM, Peters GA, et al (2011) Pyrosequencing of chaperonin-60 (*cpn60*) amplicons as a means of determining microbial community composition In: Kwon YM, Ricke SC, editors. High-throughput Next Generation Sequencing: Methods and Applications. 1 ed New York, NY: Humana Press pp. 143–158. 10.1007/978-1-61779-089-8_10 21431768

[9] Links MG, Dumonceaux TJ, Hemmingsen SM, Hill JE (2012) The chaperonin-60 universal target is a barcode for bacteria that enables *de novo* assembly of metagenomic sequence data. PLoS One 7: e49755 10.1371/journal.pone.0049755 23189159 PMC3506640

[10] Links MG, Demeke T, Gräfenhan T, Hill JE, Hemmingsen SM, et al (2014) Simultaneous profiling of seed-associated bacteria and fungi reveals antagonistic interactions between microorganisms within a shared epiphytic microbiome on *Triticum* and *Brassica* seeds. New Phytologist 202: 542–553. 10.1111/nph.12693 24444052 PMC4235306

[11] Lévesque C, Chen W, Hambleton S, Seifert K, Adam Z, et al. (2015) CRTI 09-462RD—Next generation sequencing, direct detection and genotyping of fungi, bacteria and nematodes in the agri-food system. DRDC-RDDC-2015-C148 DRDC-RDDC-2015-C148. 77 p.

[12] Hill JE, Hemmingsen SM, Goldade BG, Dumonceaux TJ, Klassen J, et al (2005) Comparison of ileum microflora of pigs fed corn-, wheat-, or barley-based diets by chaperonin-60 sequencing and quantitative PCR. ApplEnvironMicrobiol 71: 867–875. 10.1128/AEM.71.2.867-875.2005 PMC546709 15691942

[13] Schellenberg J, Links MG, Hill JE, Dumonceaux TJ, Peters GA, et al (2009) Pyrosequencing of the chaperonin-60 universal target as a tool for determining microbial community composition. Appl Environ Microbiol 75: 2889–2898. 10.1128/AEM.01640-08 19270139 PMC2681704

[14] Schoch CL, Seifert KA, Huhndorf S, Robert V, Spouge JL, et al (2012) Nuclear ribosomal internal transcribed spacer (ITS) region as a universal DNA barcode marker for Fungi. Proceedings of the National Academy of Sciences 109: 6241–6246. 10.1073/pnas.1117018109 PMC3341068 22454494

[15] Hill JE, Penny SL, Crowell KG, Goh SH, Hemmingsen SM (2004) cpnDB: A Chaperonin Sequence Database. Genome Research 14: 1669–1675. 10.1101/gr.2649204 15289485 PMC509277

[16] Pfaffl MW (2001) A new mathematical model for relative quantification in real-time RT-PCR. Nucleic Acids Res 29: e45 11328886 10.1093/nar/29.9.e45 PMC55695

[17] Tomita N, Mori Y, Kanda H, Notomi T (2008) Loop-mediated isothermal amplification (LAMP) of gene sequences and simple visual detection of products. Nat Protocols 3: 877–882. 10.1038/nprot.2008.57 18451795

[18] Burd EM (2010) Validation of Laboratory-Developed Molecular Assays for Infectious Diseases. Clinical Microbiology Reviews 23: 550–576. 10.1128/CMR.00074-09 20610823 PMC2901657

[19] Banoo S, Bell D, Bossuyt P, Herring A, Mabey D, et al (2006) Evaluation of diagnostic tests for infectious diseases: general principles. NatRevMicrobiol 4: S21–S31. 10.1038/nrmicro1523 17034069

[20] Schardl CL, Young CA, Hesse U, Amyotte SG, Andreeva K, et al (2013) Plant-Symbiotic Fungi as Chemical Engineers: Multi-Genome Analysis of the Clavicipitaceae Reveals Dynamics of Alkaloid Loci. PLoS Genet 9: e1003323 10.1371/journal.pgen.1003323 23468653 PMC3585121

[21] Links MG, Chaban B, Hemmingsen S, Muirhead K, Hill J (2013) mPUMA: a computational approach to microbiota analysis by de novo assembly of operational taxonomic units based on protein-coding barcode sequences. Microbiome 1: 23 10.1186/2049-2618-1-23 24451012 PMC3971603

[22] Tamura K, Nei M (1993) Estimation of the number of nucleotide substitutions in the control region of mitochondrial DNA in humans and chimpanzees. Mol Biol Evol 10: 512–526. 8336541 10.1093/oxfordjournals.molbev.a040023

[23] Tamura K, Stecher G, Peterson D, Filipski A, Kumar S (2013) MEGA6: Molecular Evolutionary Genetics Analysis version 6.0. Mol Biol Evol 30: 2725–2729. 10.1093/molbev/mst197 24132122 PMC3840312

[24] Comte A, Gräfenhan T, Links MG, Hemmingsen SM, Dumonceaux TJ (2016) Sequence analysis of taxonomic markers cpn60 and ITS from amplified from ergot sclerotia. Data in Brief submitted.

[25] Poinar GJ, Alderman S, Wunderlich J (2015) One hundred million year old ergot: psychotropic compounds in the Cretaceous? Palaeodiversity 8: 13–19.

[26] Magnuson BA, Patterson CA, Hucl P, Newkirk RW, Ram JI, et al (2014) Safety assessment of consumption of glabrous canary seed (Phalaris canariensis L.) in rats. Food Chem Toxicol 63: 91–103. 10.1016/j.fct.2013.10.041 24200856

[27] Alderman S, Walenta DL, Hamm PB, Martin RC, Dung JK, et al (2015) Afternoon ascospore release in *Claviceps purpurea* optimizes perennial ryegrass infection. Plant Disease 99: 1410–1415. 30690988 10.1094/PDIS-09-14-0978-RE

