# Supplementary material for: Quantitative molecular diagnostic assays of grain washes for Claviceps purpurea are correlated with visual determinations of ergot contamination
Source: PLoS One. 2017 Mar 3;12(3):e0173495. doi: 10.1371/journal.pone.0173495 (PMC5336299; doi:10.1371/journal.pone.0173495)
Supplement: S1 Fig — (PPTX) [file pone.0173495.s003.pptx]

## Slide 1
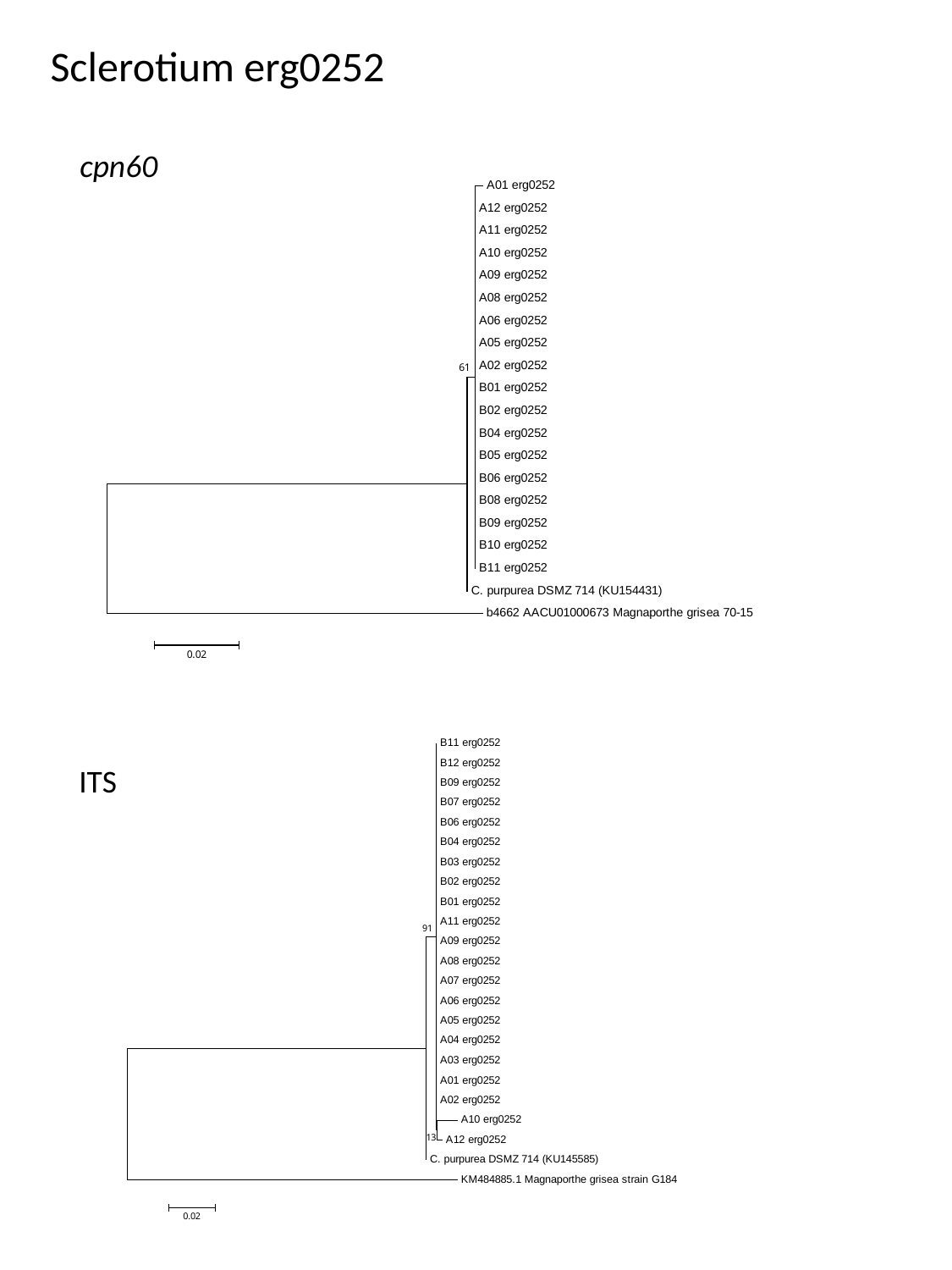

Sclerotium erg0252
cpn60
ITS

## Slide 2
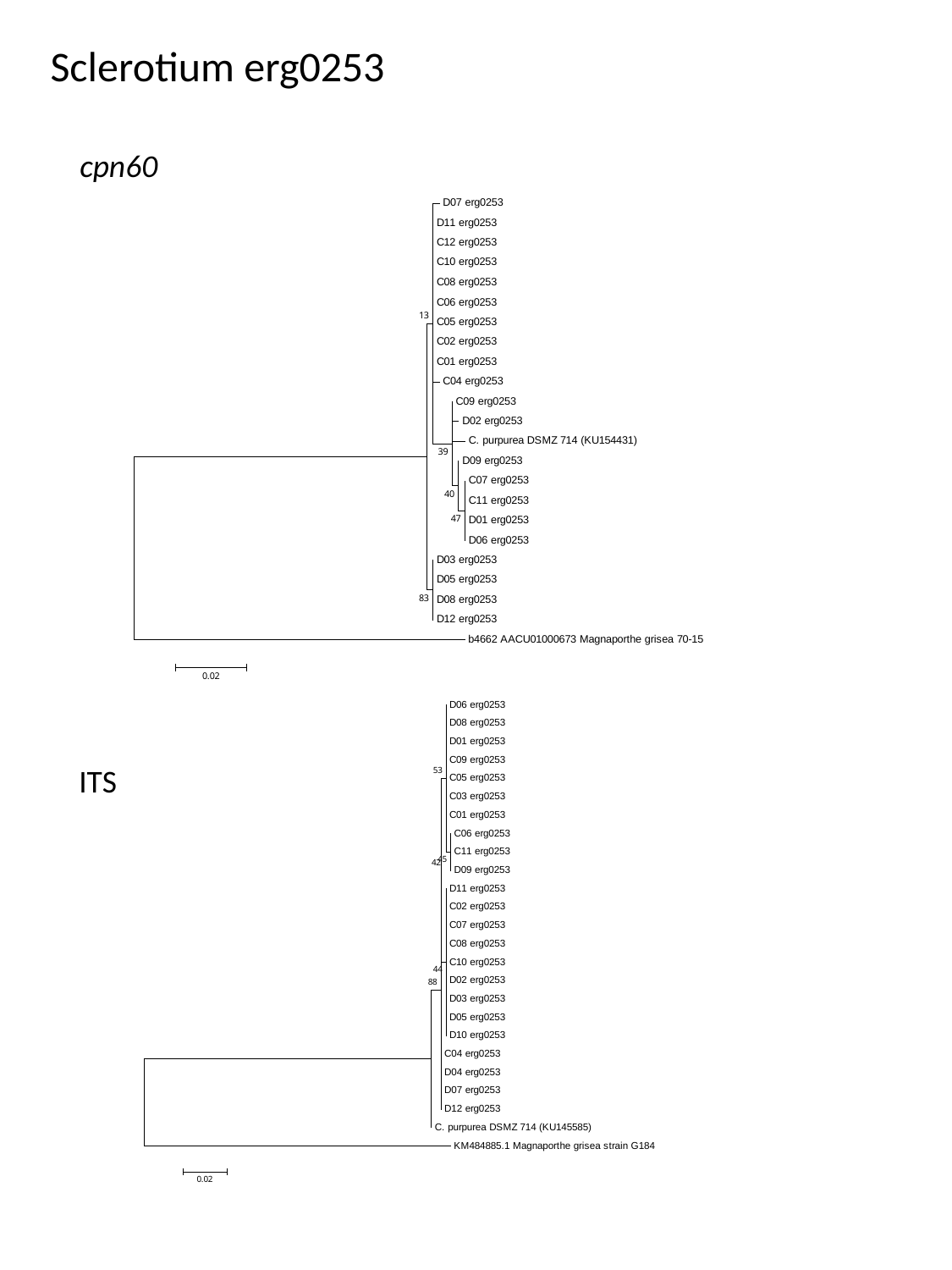

Sclerotium erg0253
cpn60
ITS

## Slide 3
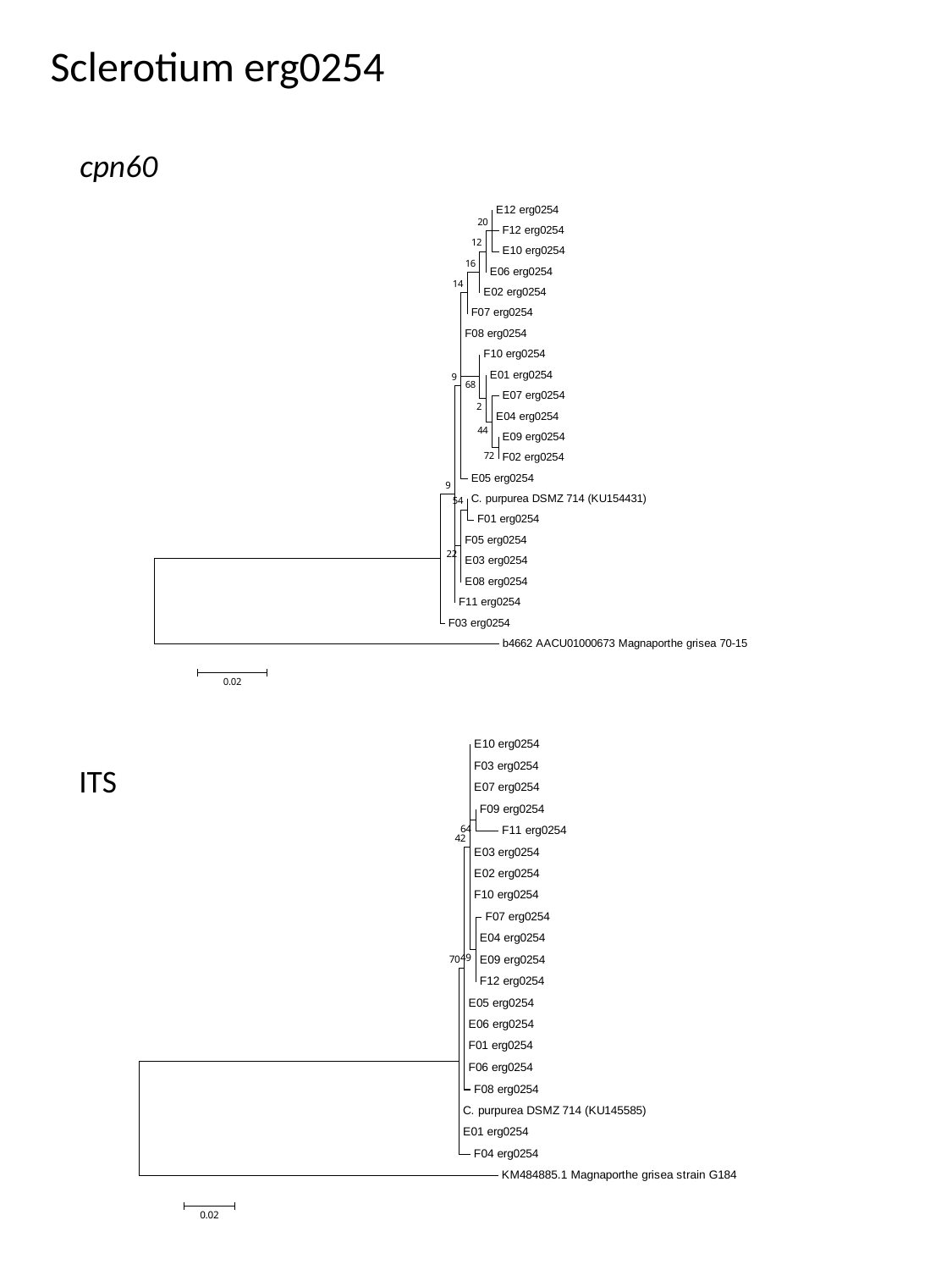

Sclerotium erg0254
cpn60
ITS

## Slide 4
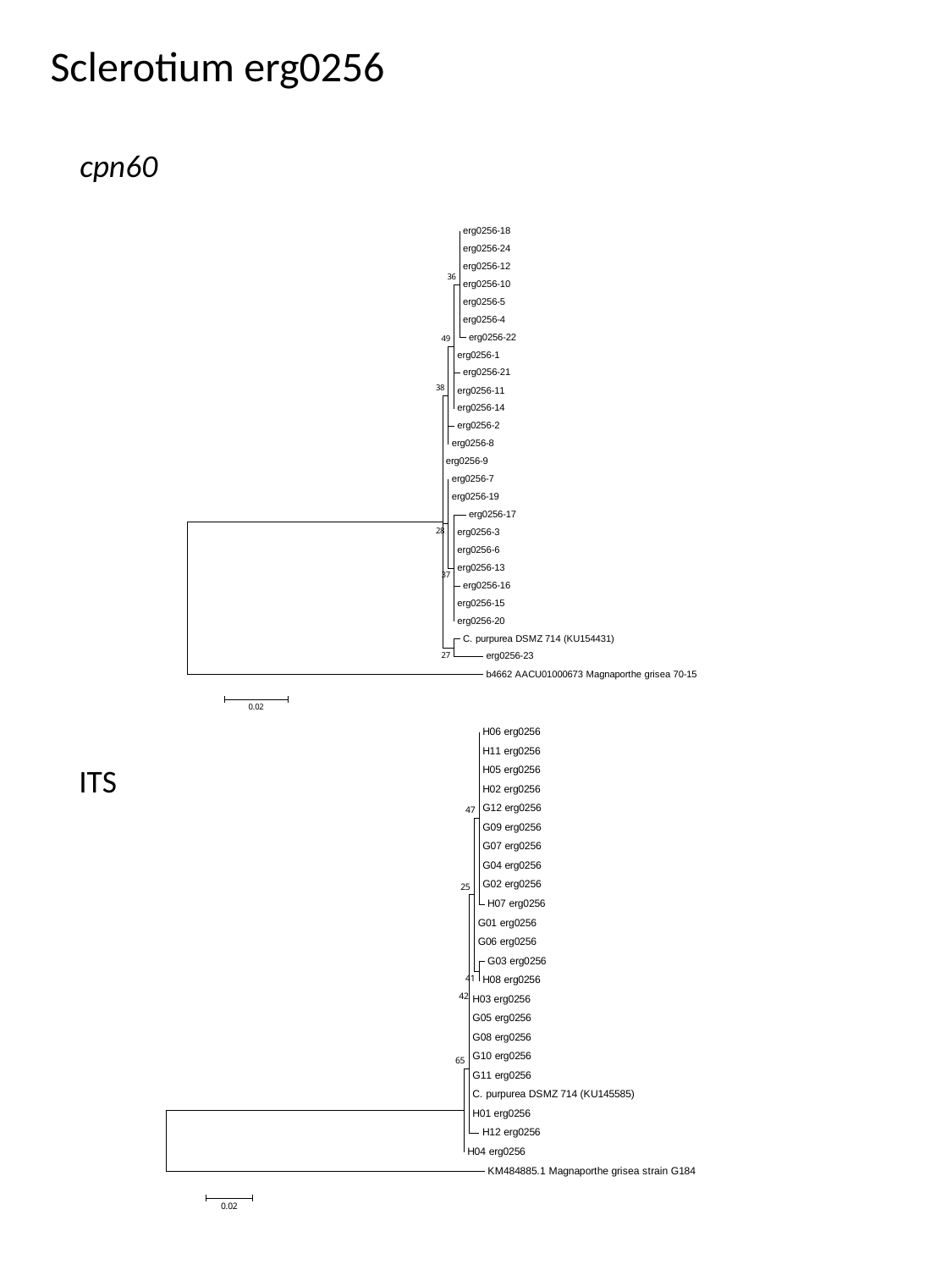

Sclerotium erg0256
cpn60
ITS

## Slide 5
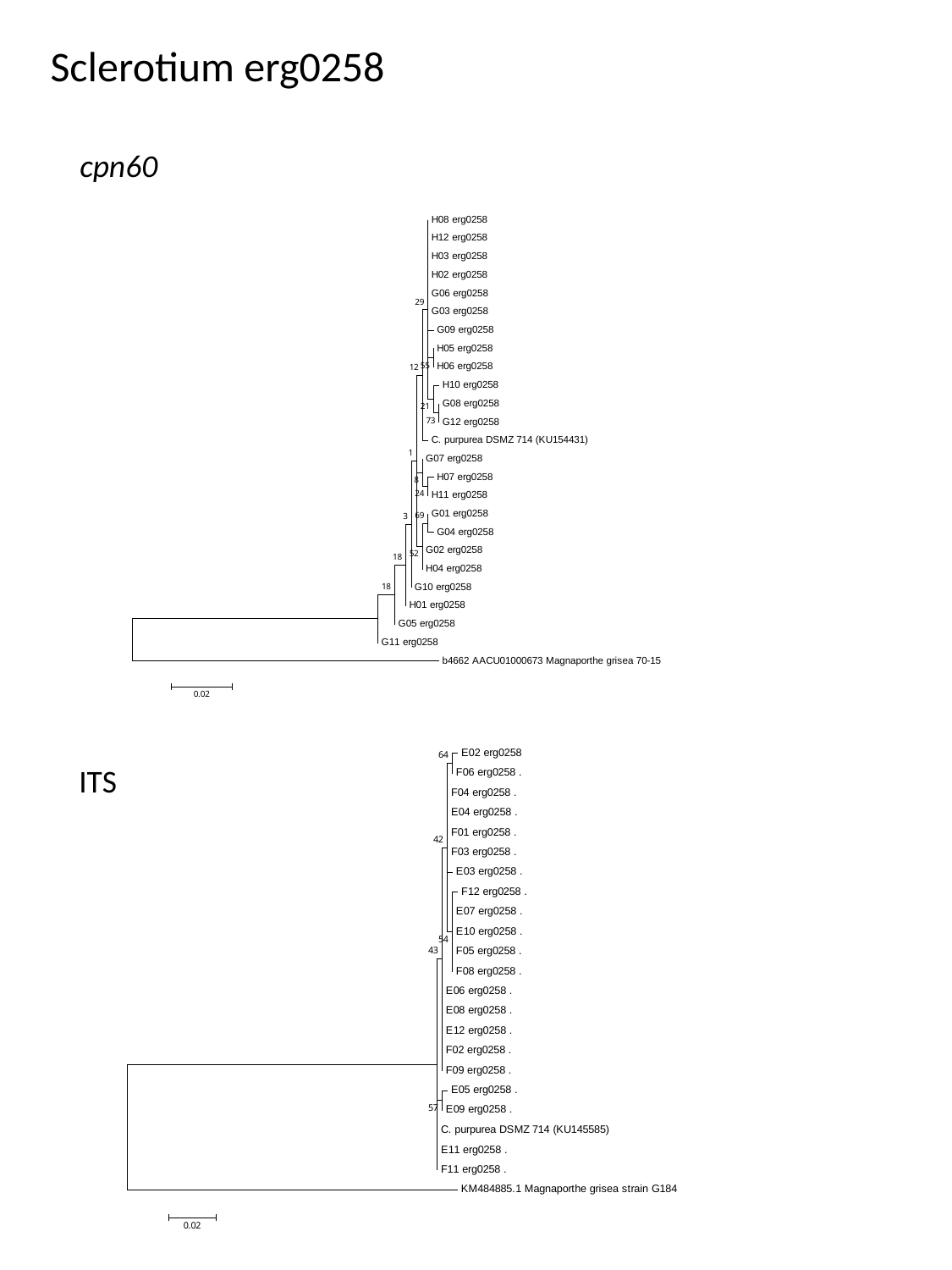

Sclerotium erg0258
cpn60
ITS
